# Mineral Content and Bioactive Potential of Quince (*Cydonia oblonga*) Peels for Value-Added Food Production

**DOI:** 10.1007/s11130-026-01528-7

**Published:** 2026-06-18

**Authors:** Alena Vollmannova, Tomas Toth, Judita Lidikova, Janette Musilova, Natalia Ceryova, Jana Jakubcinova

**Affiliations:** Institute of Food Sciences, Faculty of Biotechnology and Food Sciences, Slovak University of Agriculture in Nitra, Tr. A. Hlinku 2, Nitra, 94976 Slovakia

**Keywords:** Quince, Chlorogenic acid, Rutin, Mineral composition

## Abstract

The food sector is resource-intensive and under pressure to reduce its environmental footprint, with by-product valorization offering a sustainable solution. This study evaluated the potential of quince peels, a major processing by-product, for innovative food applications. Nine quince varieties from an organic farm in the Czech Republic were analyzed over three years. Elemental composition was determined by AAS, total polyphenols and antioxidant activity spectrophotometrically, and phenolics by HPLC. Quince peels were rich in K (4096–8068 mg/kg), Ca (701–1789 mg/kg), Mg (447–1201 mg/kg), and P (588–1544 mg/kg), with low Na content.The contents of Cu and Zn ranged from 3.2 to 9.6 and 6.9–27.2 mg/kg, respectively. Also, levels of Mn, Fe, Cr, Ni and Co (0.9–2.6; 17.7–56.1; 0.1–1.0; 0.1–2.1 and < LOD – 0.8 mg/kg, respectively) were remarkable. The contents of Pb and Cd were below the limit of detection in most samples. The average total phenolic content in quince peels was 13.82 mg GAE/g DW. Antioxidant activity varied depending on the assay, with high reducing power (FRAP), moderate radical scavenging activity (DPPH), and lower ABTS values. On the other hand, quince peels are an excellent source of chlorogenic acid (5-CQA) which average value was 2870 mg/kg DW, and its derivatives, such as neochlorogenic acid (3-CQA), cryptochlorogenic acid (4-CQA) as well as 3,5-dicaffeoylquinic acid (3,5-DCQA). The surprising component of the quince peel was rutin (average content 3940 mg/kg DW). These findings suggest that quince peels represent a promising ingredient for the development of functional food products, particularly as natural mineral and antioxidant-rich additives in bakery products, fruit-based preparations, and meat formulations.

**Supplementary Information:**

The online version contains supplementary material available at https://doi.org/10.1007/s11130-026-01528-7.

## Introduction

 The food sector is the most resource-intensive sector and does not fully respect the Union’s fundamental principles on waste management set out in the waste hierarchy, which require prioritising waste prevention, followed by preparing for re-use and recycling. It is under increasing pressure to reduce its ecological footprint. In the EU, over 58 million tonnes of food waste (130 kg/inhabitant) are generated annually, with an associated market value estimated at 132 billion euros. At the same time, over 38 million people cannot afford a quality meal every second day [1].

The food industry is an important part of the global economy and a significant contributor to environmental problems. Sustainability in food systems involves implementing environmentally friendly practices that reduce carbon emissions, water consumption and waste.

One way to achieve sustainability in food systems is to transform by-products into valuable resources that can be used in innovative foods. Fresh fruits and vegetables are highly utilized commodities in the food industry. However, their processing generates significant amounts of waste, which, despite their remarkable content of biologically valuable components, creates serious economic and environmental problems. It is the content composition of by-products of fruit and vegetable processing that has stimulated research activities in terms of their use for the development of enriched and functional foods and thus contribute to sustainable resource management. Currently, research supporting the use of plant-based preservatives, especially fruit peel extracts, in the food industry is gaining attention. There are scientific studies confirming that some by-products generated during fruit processing, such as waste from fruit and vegetable peels, have the potential to be used in the functional food industry to produce value-added products [2–4].

The fruit of the quince (*Cydonia oblonga* Mill.) that belongs to the *Rosaceae* family is considered unusual and even forgotten, but today it is again coming back to attention as a rediscovered fruit species. Quince began to be cultivated in Transcaucasia around 4,000 BC [5]. It is an extremely resistant and tolerant fruit species. Thanks to these properties it has spread from Transcaucasia in the east to China, and in the west to Europe and the Mediterranean region. The ancient Greeks and Romans considered quince trees sacred. Venus, the ancient Roman goddess of spring and beauty, was often depicted in paintings and sculptures with a quince fruit in her right hand. Over time, the Greek goddess Aphrodite, was also depicted with the quince in her hand, too. In ancient Greece, quince fruits were considered a symbol of love and happiness. Quince is a rich source of macroelements and microelements such as potassium, phosphorous, magnesium, calcium, iron, copper, and zinc. The fruit is also characterized by a diverse composition of bioactive compounds and nutrients. Quince pulp contains 81.20% moisture, while other physicochemical components include proteins (0.50–0.71 g/100 g), lipids (0.21–2.4 g/100 g), fibers (1.10–2.6%), starch (7.30 g/100 g), ashes (0.53–0.70 g/100 g), total sugars (10.90 g/100 g), vitamin C (15.32 g/100 g), and total pectin (12.67 g/100 g). The peel and pulp are also rich in phytochemicals such as quercetin, 5-*O*-caffeoylquinic acid, rutin, *β*-carotene, lycopene, *β*-cryptoxanthin, lutein, and zeaxanthin, which are associated with high antioxidant potential and may contribute to the prevention and treatment of various pathologies [6]. The low-fat fruit is also rich in antioxidants that have significant positive effects on health. The health benefits of quince fruit consumption have been linked to its hypoglycemic, anti-inflammatory, antibacterial, and anticancer properties [7, 8]. Ripe quince fruits have a pleasant, long-lasting and strong taste. However, due to its bitterness, quince is underutilized [9]. They are in great demand for processing marmalades, jams, jellies and cakes [10].

In addition to the main products, quince fruit processing also generates by-products, *i.e*., fruit pressings consisting of peels, seeds, and pulp residues. Although these processing residues are commonly used in agriculture, current sustainability strategies and circular economy approaches promoted by organizations such as the United Nations Environment Programme, the European Commission, FAO, and the World Resources Institute emphasize their reintegration into the human food chain due to their favorable chemical composition [11]. Therefore, the aim of this study was to assess the potential utilization of quince peels, as the main by-product of quince fruit processing, in innovative food products.

Despite increasing interest in fruit-processing by-products as sources of bioactive compounds, comprehensive studies evaluating the multiyear variability of mineral composition, phenolic profile, and antioxidant activity of quince peel across different cultivars remain limited. Furthermore, integrated studies combining macroelements, trace elements, toxic elements, antioxidant activity, and individual phenolic compounds in quince peel are still scarce. Therefore, the aim of this study was to comprehensively characterize the mineral composition, antioxidant activity, and phenolic profile of quince peel obtained from technologically processed fruits cultivated over a three-year period.

## Materials and Methods

Detailed procedures for analytical determinations and statistical analysis used are provided in the supplementary materials.

## Results and Discussion

### Determination of Macroelements Content

Based on our results (Table 1), it can be stated that quince peels are a significant source of K (4096–8068 mg/kg), which is higher than potassium content in avocados, kiwifruit, or bananas [12]. On the other hand, quince peels have a low content of Na (17–78 mg/kg) comparable to plums, peaches, apricots [13] or raspberry fruits [14]. The quince peels are also rich in Ca (701–1789 mg/kg), comparable to spinach [15], Mg (447–1201 mg/kg), and P (588–1544 mg/kg).

**Table 1:** Average values of macroelement contents in quince peel (mg/kg)

Quince variety	Year	K	Na	Ca	Mg	*P*
Aurelia	2021	5717^a^	58.30^c^	990^b^	1003^b^	1263^a^
Aurelia	2022	8068^b^	42.00^b^	727^a^	1122^c^	1444^b^
Aurelia	2023	7344^b^	31.7^a^	1195^c^	659^a^	1244^a^
*Cydora Robusta*	2021	4807^a^	38.80^a^	1789^c^	792^b^	911^ab^
*Cydora Robusta*	2022	6934^c^	47.10^b^	1426^b^	1143^c^	957^b^
*Cydora Robusta*	2023	5707^b^	36.7^a^	948^a^	611^a^	833^a^
Hruškovitá	2021	6365^ab^	51.30^c^	1144^ab^	913^b^	1196^b^
Hruškovitá	2022	7151^b^	42.80^b^	1239^b^	1196^c^	976^a^
Hruškovitá	2023	5831^a^	37.1^a^	1070^a^	583^a^	1066^ab^
Izobilnaja	2021	5419^a^	41.20^ab^	1067^a^	836^b^	1132^b^
Izobilnaja	2022	6083^a^	70.60^b^	1412^b^	1112^c^	789^a^
Izobilnaja	2023	5872.6^a^	37.1^a^	1136^a^	608^a^	1233^b^
Kocúrova	2021	5003^b^	65.00^b^	1648^b^	931^a^	1031^c^
Kocúrova	2022	5736^c^	54.60^ab^	1511^b^	966^a^	723^b^
Kocúrova	2023	4096^a^	16.8^a^	971^a^	447^a^	588^a^
Konstantinopolska	2021	5069^a^	72.20^c^	1604^a^	722^b^	870^b^
Konstantinopolska	2022	6299^b^	63.00^b^	1671^a^	837^c^	723^a^
Konstantinopolska	2023	5364^a^	35.7^a^	1059^a^	488^a^	788^ab^
Morava	2021	6465^a^	44.50^ab^	1021^c^	1125^a^	1277^b^
Morava	2022	6228^a^	77.90^b^	701^a^	1156^a^	1070^a^
Morava	2023	7607^b^	36.9^a^	889^b^	732^a^	1633^c^
Otličnica	2021	5205^a^	23.50^b^	1496^a^	1036^b^	1065^a^
Otličnica	2022	6734^b^	35.40^c^	984^a^	1081^b^	1013^a^
Otličnica	2023	7534^b^	19.8^a^	1019^a^	869^a^	1544^a^
Plovdivskaja	2021	5416^a^	31.90^a^	1424^c^	1075^b^	971^a^
Plovdivskaja	2022	6897^b^	32.40^a^	1200^b^	1201^b^	948^a^
Plovdivskaja	2023	7101^b^	32.7^a^	1028^a^	769^a^	1322^a^

*Different letters indicate statistically significant differences (*p* < 0.05) among years within each cultivar, as determined by appropriate statistical tests

From a nutritional perspective, quince peel may contribute to the dietary intake of several essential mineral elements. Considering the average potassium content (approximately 4095–8068 mg/kg DW), incorporation of 10 g of dried quince peel powder into a food product could provide approximately 40–80 mg of potassium, corresponding to about 1–2% of the adequate daily intake for adults (3500 mg/day). Similarly, quince peel may represent a supplementary source of calcium, magnesium, and phosphorus, although its contribution to the total daily intake would remain moderate under realistic consumption scenarios.

**Table 2:** Results of two-way analysis of variance with effect size estimates evaluating the effects of cultivar, year, and their interaction on macroelement composition of quince peel

Variable	Factor	F	*p*-value	η²	ω²
K	Cultivar	40.75	< 0.0001	0.400	0.390
	Year	101.00	< 0.0001	0.248	0.245
	C × Y	14.57	< 0.0001	0.286	0.266
Na	Cultivar	195.90	< 0.0001	0.354	0.352
	Year	711.72	< 0.0001	0.321	0.321
	C × Y	86.79	< 0.0001	0.313	0.310
Ca	Cultivar	96.05	< 0.0001	0.402	0.398
	Year	167.50	< 0.0001	0.175	0.174
	C × Y	47.08	< 0.0001	0.394	0.386
Mg	Cultivar	69.85	< 0.0001	0.221	0.218
	Year	852.60	< 0.0001	0.674	0.673
	C × Y	13.29	< 0.0001	0.084	0.078
P	Cultivar	126.70	< 0.0001	0.571	0.566
	Year	75.43	< 0.0001	0.085	0.084
	C × Y	34.87	< 0.0001	0.314	0.305

*Degrees of freedom were identical for all variables (cultivar: 8; year: 2; interaction: 16). η² and ω² represent effect size estimates.

Two-way ANOVA revealed that both cultivar and year significantly affected the mineral composition of quince peel samples (*p* < 0.0001 for all factors), with varying contributions of each factor depending on the element (Table 2). For potassium (K), variability was strongly influenced by cultivar (η² = 0.400), followed by interaction effects (η² = 0.286) and year (η² = 0.248), indicating both genetic control and environmental modulation. Sodium (Na) showed a more balanced contribution of all factors, with cultivar (η² = 0.354), year (η² = 0.321), and their interaction (η² = 0.313) contributing similarly to total variability. In contrast, calcium (Ca) variability was predominantly driven by cultivar (η² = 0.402) and interaction effects (η² = 0.394), while the effect of year was less pronounced (η² = 0.175). Magnesium (Mg) exhibited a distinct pattern, where year was the dominant factor (η² = 0.674), indicating a strong environmental influence, whereas cultivar (η² = 0.221) and interaction (η² = 0.084) played smaller roles. For phosphorus (P), variability was mainly explained by cultivar (η² = 0.571), with a substantial contribution from interaction effects (η² = 0.314), while year had a relatively minor influence (η² = 0.085). Overall, these results demonstrate that the relative importance of genotype (cultivar), environment (year), and their interaction differ among elements, with some elements being predominantly genotype-driven (e.g., P, Ca), others strongly influenced by environmental conditions (e.g., Mg), and several showing combined genotype–environment effects.

### Determination of Microelements and Heavy Metals Content

The contents of Cu and Zn ranged from 3.2 to 9.6 and 6.9–27.2 mg/kg, respectively). The zinc content in quince peels is especially interesting and is significantly higher than Zn in avocados [16] or in apples, plums, raspberries, red currants and black currants [17]. Also, levels of Mn, Fe, Cr, Ni and Co (0.9–2.6; 17.7–56.1; 0.1–1.0; 0.1–2.1 and < LOD – 0.8 mg/kg, respectively) are remarkable. Iron in particular deserves attention, the content of which in quince peels is higher than in apples or blueberries [18] and comparable to the Fe content in cranberries [19]. Quince peels as a component of innovative foods therefore appear to be a promising source of iron in the diet and may contribute to reducing Fe deficiency in the human body, which is very often observed nowadays. The contents of Pb and Cd were below the limit of detection (LOD) in most samples (Table 3). These results indicate a low risk associated with toxic element content and at the same time point out the health benefits of quince peels as a source of important minerals, especially zinc and iron, in the production of functional foods. The nutritional relevance of trace elements appears particularly interesting in the case of iron and zinc. Based on the determined concentrations, 10 g of dried quince peel powder could provide approximately 0.18–0.56 mg Fe and 0.07–0.27 mg Zn. This corresponds to approximately 1–7% of the recommended daily intake for iron (8–18 mg/day) and about 1–3% of the recommended intake for zinc (8–11 mg/day), depending on sex and physiological status. Although quince peel cannot be considered a primary dietary source of these elements, its incorporation into innovative food formulations may contribute to the overall nutritional value of enriched products. Importantly, the generally low concentrations of Pb and Cd indicate minimal toxicological concern regarding the potential dietary use of quince peel under the investigated cultivation conditions.

**Table 3:** Average values of microelements and risk elements contents in quince peel (mg/kg)

Quince variety	Year	Cu	Zn	Mn	Fe	Cr	Ni	Co	Pb	Cd
Aurelia	2021	5.20^a^	8.20^a^	1.30^a^	25.80^a^	0.30^ab^	0.10^a^	0.10^a^	< LOD^a^	0.20^b^
Aurelia	2022	8.40^ab^	13.70^b^	1.30^a^	24.90^a^	1.00^b^	2.00^c^	0.70^a^	< LOD^a^	< LOD^a^
Aurelia	2023	9.1^b^	21.3^c^	1.30^a^	43.3^b^	0.1^a^	1.10^b^	0.1^a^	0.3^b^	< LOD^a^
Cydora R.	2021	4.40^b^	6.90^a^	2.30^c^	17.70	0.30^a^	0.40^a^	0.40	< LOD	< LOD^a^
Cydora R.	2022	3.80^a^	16.40^b^	1.70^b^	18.50	1.00^b^	2.00^c^	0.60	0.80^b^	0.10^b^
Cydora R.	2023	3.8^a^	15.6^b^	1.4^a^	25.1	0.3^a^	1.50^b^	< LOD	0.8^b^	< LOD^a^
Hruškovitá	2021	6.60^b^	16.10^a^	1.60^ab^	29.50^a^	0.10^a^	0.40^a^	0.60^a^	< LOD^a^	< LOD^a^
Hruškovitá	2022	7.30^b^	16.60^a^	2.40^b^	39.80^b^	1.00^b^	1.70^b^	0.60^a^	1.30^b^	0.13^b^
Hruškovitá	2023	5.7^a^	19.6^b^	1.4^a^	31.5^a^	0.2^ab^	0.8^ab^	< LOD^a^	< LOD^a^	< LOD^a^
Izobilnaja	2021	4.50^a^	8.40^a^	1.80^a^	24.60^a^	0.30^ab^	0.40^a^	0.40^b^	< LOD^a^	< LOD^a^
Izobilnaja	2022	4.30^a^	11.30^b^	2.20^b^	29.40^b^	0.90^b^	1.50^b^	0.70^c^	0.40^ab^	0.19^b^
Izobilnaja	2023	6.2^a^	18.8^c^	1.80^a^	32.4^b^	0.2^a^	1.4^ab^	< LOD^a^	0.5^b^	0.01^ab^
Kocúrova	2021	6.70^c^	10.90^c^	2.00^b^	27.90^b^	0.40^ab^	0.30^a^	0.10^ab^	0.14^b^	0.30^c^
Kocúrova	2022	3.20^a^	7.90^a^	2.60^c^	25.30^b^	0.90^b^	1.90^b^	0.60^b^	0.30^c^	0.16^b^
Kocúrova	2023	4.4^b^	9.6^b^	1.00^a^	21.4^a^	0.2^a^	0.5^ab^	< LOD^a^	< LOD^a^	< LOD^a^
Konstantinopolska	2021	5.20^a^	9.60^a^	1.80^b^	22.50^a^	0.10^a^	0.30^a^	0.10^ab^	< LOD^a^	< LOD^a^
Konstantinopolska	2022	4.60^a^	8.70^a^	2.00^b^	22.60^a^	1.00^c^	2.10^c^	0.50^b^	< LOD^a^	< LOD^a^
Konstantinopolska	2023	6.1^b^	11.4^b^	1.3^a^	22.3^a^	0.5^b^	1^b^	< LOD^a^	< LOD^a^	0.06^b^
Morava	2021	8.80^b^	16.30^a^	1.50^b^	45.90^a^	0.50^b^	0.30^a^	0.40^b^	< LOD^a^	0.10^b^
Morava	2022	7.60^a^	20.60^b^	1.10^a^	23.80^a^	0.80^c^	1.50^b^	0.50^c^	< LOD^a^	< LOD^a^
Morava	2023	9.6^b^	27.2^c^	1.5^b^	48.3^a^	0.3^a^	1.4^ab^	0.2^a^	< LOD^a^	< LOD^a^
Otličnica	2021	5.20^b^	11.20^ab^	1.50^b^	25.70^ab^	0.10^a^	0.10^a^	0.10^a^	0.02^b^	0.20^b^
Otličnica	2022	3.80^a^	9.90^a^	1.00^a^	23.80^a^	1.00^b^	1.00^b^	0.80^b^	< LOD^a^	< LOD^a^
Otličnica	2023	7.3^c^	20.1^b^	1.4^b^	56.1^b^	0.1^a^	0.7^ab^	0.1^a^	< LOD^a^	< LOD^a^
Plovdivskaja	2021	5.00^a^	8.40^a^	1.60^a^	26.60^a^	0.50^b^	0.40^a^	0.50^b^	< LOD^a^	< LOD^a^
Plovdivskaja	2022	7.00^b^	13.00^b^	1.60^a^	27.70^a^	0.80^c^	1.70^c^	0.50^b^	0.40^b^	0.15^c^
Plovdivskaja	2023	8.7^c^	18.6^c^	0.9^a^	35.5^b^	0.3^a^	1.2^b^	0.20^a^	< LOD^a^	0.07^b^

*Different letters indicate statistically significant differences (*p* < 0.05) among years within each cultivar, as determined by appropriate statistical tests

**Table 4:** Two-way ANOVA and effect size estimates for the influence of cultivar, year, and their interaction on microelement content in quince peel

Variable	Factor	F	*p*-value	η²	ω²
Cu	Cultivar	222.54	< 0.0001	0.584	0.581
	Year	125.86	< 0.0001	0.083	0.082
	C × Y	60.19	< 0.0001	0.316	0.310
Zn	Cultivar	229.52	< 0.0001	0.416	0.414
	Year	755.70	< 0.0001	0.342	0.342
	C × Y	63.21	< 0.0001	0.229	0.226
Mn	Cultivar	94.95	< 0.0001	0.329	0.325
	Year	228.94	< 0.0001	0.198	0.197
	C × Y	64.93	< 0.0001	0.450	0.443
Fe	Cultivar	143.25	< 0.0001	0.432	0.429
	Year	220.78	< 0.0001	0.166	0.166
	C × Y	63.18	< 0.0001	0.381	0.375
Cr	Cultivar	62.79	< 0.0001	0.035	0.034
	Year	6041.80	< 0.0001	0.831	0.831
	C × Y	118.91	< 0.0001	0.131	0.130
Ni	Cultivar	147.07	< 0.0001	0.079	0.079
	Year	6219.73	< 0.0001	0.836	0.836
	C × Y	75.47	< 0.0001	0.081	0.080
Co	Cultivar	161.74	< 0.0001	0.072	0.072
	Year	6586.67	< 0.0001	0.735	0.735
	C × Y	212.53	< 0.0001	0.190	0.189
Pb	Cultivar	1688.22	< 0.0001	0.329	0.329
	Year	3936.13	< 0.0001	0.192	0.192
	C × Y	1225.37	< 0.0001	0.478	0.477
Cd	Cultivar	557.45	< 0.0001	0.178	0.177
	Year	1852.47	< 0.0001	0.147	0.147
	C × Y	1056.31	< 0.0001	0.673	0.672

*Degrees of freedom were identical for all variables (cultivar: 8; year: 2; interaction: 16). η² and ω² represent effect size estimates

Two-way ANOVA revealed that the variability of trace element concentrations in quince peel was significantly influenced by cultivar, year, and their interaction (*p* < 0.0001 for all factors), with the relative contribution of each factor differing among elements (Table 4). Copper (Cu), zinc (Zn), and iron (Fe) were predominantly affected by cultivar (η² = 0.416–0.584), indicating strong genetic control over their accumulation. Nevertheless, year also contributed substantially to variability in Zn (η² = 0.342) and Fe (η² = 0.166), suggesting that environmental conditions play a secondary but relevant role. Manganese (Mn) exhibited a more balanced pattern, with considerable contributions from both cultivar (η² = 0.329) and interaction effects (η² = 0.450), indicating that its accumulation is strongly modulated by the combined influence of genotype and environmental conditions. In contrast, chromium (Cr), nickel (Ni), and cobalt (Co) were predominantly driven by year (η² = 0.735–0.836), reflecting a strong environmental influence with only minor contributions from cultivar. For toxic elements, distinct patterns were observed. Lead (Pb) showed substantial contributions from both cultivar (η² = 0.329) and interaction effects (η² = 0.478), indicating that its accumulation is influenced by both genetic and environmental factors. Cadmium (Cd), however, was primarily governed by interaction effects (η² = 0.673), suggesting a strong dependence on the specific combination of cultivar and growing conditions. It should be noted that, despite data transformation, selected trace elements (Pb, Cd, Cr, Ni, and Co) did not fully meet the assumption of normality. Therefore, these results should be interpreted primarily in terms of variability structure rather than precise statistical inference. The findings indicate that trace element accumulation in quince peel is controlled by a complex interplay of genetic and environmental factors, with element-specific patterns ranging from genotype-dominated (Cu, Zn, Fe) to environment-driven (Cr, Ni, Co) and interaction-dependent behavior (Mn, Pb, Cd).

The variability observed in the mineral composition of quince peel could result from a complex interaction between genetic and environmental factors. The strong cultivar effects observed for several macroelements, and microelements suggest substantial genotype-dependent regulation of nutrient uptake, transport, and accumulation. In contrast, the stronger influence of year on magnesium and selected trace elements indicates a significant environmental contribution, likely associated with climatic conditions, soil moisture, soil physicochemical properties, and nutrient bioavailability [20]. The significant cultivar × year interactions further demonstrate that individual cultivars responded differently to environmental conditions during specific growing seasons, highlighting genotype-dependent environmental adaptability in mineral accumulation (Table 5).

**Table 5:** Correlation matrix of elements (Spearman)

Variables	K	Na	Ca	Mg	P	Cu	Zn	Mn	Fe	Cr	Ni	Co	Pb	Cd
K	**1**													
Na	-0.09	**1**												
Ca	-0.36	0.25	**1**											
Mg	0.37	0.31	0.07	**1**										
P	**0.58**	-0.13	**-0.48**	0.10	**1**									
Cu	**0.54**	-0.11	-0.33	0.07	**0.73**	**1**								
Zn	**0.67**	-0.18	**-0.44**	-0.03	**0.57**	**0.68**	**1**							
Mn	-0.21	**0.47**	**0.77**	0.20	-0.37	-0.30	-0.35	**1**						
Fe	**0.54**	-0.19	-0.18	0.06	**0.65**	**0.65**	**0.61**	-0.02	**1**					
Cr	0.27	0.36	0.03	**0.63**	-0.29	-0.21	-0.18	0.20	-0.28	**1**				
Ni	**0.55**	0.12	-0.08	0.23	-0.21	-0.05	0.29	0.12	-0.07	**0.63**	**1**			
Co	**0.44**	0.36	0.13	**0.77**	-0.04	-0.13	-0.15	0.28	-0.09	**0.69**	**0.42**	**1**		
Pb	0.11	0.04	0.31	0.23	-0.26	-0.18	0.18	**0.43**	0.15	0.20	**0.43**	0.07	**1**	
Cd	-0.10	0.22	0.33	**0.40**	-0.09	-0.04	-0.15	0.29	0.17	0.23	-0.06	0.06	**0.51**	**1**

*Values in bold are different from 0 with a significance level alpha = 0.05

Spearman correlation analysis revealed pronounced relationships among mineral elements, indicating coordinated accumulation patterns as well as potential antagonistic interactions. Potassium showed strong positive correlations with zinc, phosphorus, copper, iron, nickel, and cobalt. Phosphorus exhibited strong positive correlations with copper, iron, and zinc. Similarly, copper and zinc were strongly correlated. A particularly strong association was observed between calcium and manganese. Magnesium showed strong positive correlations with cobalt and chromium. In addition, chromium was strongly correlated with nickel and cobalt. Among potentially toxic elements, such as lead and cadmium, lead showed moderate positive correlations with manganese and nickel, while cadmium was associated with lead, indicating possible co-accumulation and shared environmental sources.

### Determination of Total Polyphenols, Antioxidant Activity and Selected Phenolics Content

The average total phenolic content in quince peels was 13.82 mg GAE/g DW and the average antioxidant activity value 10.02, 140.56 and 0.28 µmol TE/g DW determined by DPPH, FRAP and ABTS methods, respectively. The TPC values in quince peels were comparable to TPC in plums [21] or berries [22]. Vieira et al. [23] determined higher total phenolic contents in the peels of three apple cultivars compared to TPC in flesh or in the whole apple fruit. Despite of this fact, the determined values were significantly lower (1.41–2.15 mg GAE/g DW) than those in the peel (6.31–32.28 mg GAE/g DW) of our investigated quince cultivars (Table 3). Apples were used as a reference due to their taxonomic relationship with quince, as both species belong to the Rosaceae family. On the contrary, the AA values determined using ABTS method were significantly higher (2.10 µmol TE/g FW) in apple peels compared to the AA in quince peels (0.13–0.55 µmol TE/g DW, corresponding 0.03–0.13 µmol TE/g FW). Another study published by Sadef et al [24] reports significant higher values of TPC in apple peels (228 mg GAE/g) and in mango peels even 503 mg GAE/g. Chan-León et al [25] studied three varieties of papaya, in which they determined the values of total polyphenol content in the peels of the fruits (7.5–9.1 mg GAE/g DW) comparable to the values determined in the peels of our quince varieties. On the other hand, the values of AA determined by DPPH method in peels of papaya (0.8–1.7 µmol TE/g DW) were approximately tenfold lower compared to values in quince peels. The studies performed by Fawole et al. [26] on peels of seven pomegranate fruit cultivars commercially grown in South Africa confirmed higher TPC values (179–295 mg GAE/g DW). The total phenolic content is determined by several factors, primarily the variety and climatic conditions of fruit cultivation.

On the other hand, quince peels were an excellent source of chlorogenic acid (5-CQA), which average value was 2870 mg/kg DW, and its derivatives, such as neochlorogenic acid (3-CQA), cryptochlorogenic acid (4-CQA) as well as 3,5-dicaffeoylquinic acid (3,5-DCQA). Awad et al. [27] determined 0.20 mg 5-CQA/g DW in skin of two apple varieties, which is approximately tenfold lower than 5-CQA content in the examined quince peels. A wide variation in chlorogenic acid content in cultivated and wild apples was determined by Liao et al [28], who who observed that the average CA content was significantly higher in cultivated apples (469.18 µg/g DW) than that in wild apples (141.26 µg/g DW). According to the study realised by Lončarić et al [29] the most abundant polyphenol in traditional apple peel was chlorogenic acid (1143 µg/g DW), which is only about 40% of the average CA content determined in peel of our quince varieties. The quince peel seems to be the more valuable source of 5-CQA compared to apple fruit as well as apple peel.

Similar findings were reported by Pontes et al [30], who identified 5-CQA as the major chlorogenic acid present in most of the tropical fruits studied. On the basis of the 5-CQA content found in the pulp, 15 of the fruits were classified as follows: very low concentration (4.4–15.8 mg/kg), low concentration (28.9–66.4 mg/kg), medium concentration (132 mg/kg), high concentration (473–474 mg/kg) or very high concentration (1730 mg/kg). The determined 5-CQA content was variable, being significantly higher in the peel of 10 fruit samples. One of the analysed fruit species was also Cydonia oblonga, in which the 5-CQA content in peel was 1507 mg/kg DW, being approximately 50% lower compared to our average value.

Our results show that quince peels are an excellent source of chlorogenic acid which is very beneficial for human health due to its neuroprotection for neurodegenerative disorders and diabetic peripheral neuropathy, anti-inflammation, anti-oxidation, anti-pathogens, mitigation of cardiovascular disorders, skin diseases, diabetes mellitus, liver and kidney injuries, and anti-tumour activities [31].

The surprising component of the quince peel is rutin (Table 6). Our findings are comparable to the results of the study realised by Hanan et al. [32], who reported the average quantity of rutin in quince peel to be 4.9 µg/mg. These findings are supported also by Silva et al [33], who investigated industrial and traditional quince jams. Data show that industrial quince jams had higher rutin content than the traditional ones which indicates the presence of a higher proportion of quince peel in these jams. Rutin is characterized by several proven positive effects on the human body, with multispectral pharmacological benefits for the treatment of various chronic diseases, such as cancer, diabetes, hypertension, and hypercholesterolemia [34]. Thanks to these biologically effective components, chlorogenic acid and rutin, quince peels represent valuable raw material with the potential for use in innovative food products.

**Table 6:** Average values of total polyphenol content (TPC), antioxidant activity (AA), content of neochlorogenic acid (NCA), chlorogenic acid (CA), cryptochlorogenic acid (CCA), 3.5 dicaffeoyl acid (DCA), and rutin in quince peel

Quince variety	Year	TPC (mg GAE/g DW	DPPH (mmol TE/kg DW)	FRAP (mmol TE/kg DW)	ABTS (mmol TE/kg DW)	NCA (mg/kg DW)	CA (mg/kg DW)	CCA (mg/kg DW)	DCA (mg/kg DW)	Rutin (mg/kg DW)
Aurelia	2021	14.80^c^	9.30^a^	79.65^a^	0.49^c^	1872^b^	4392^c^	376^c^	92.02^c^	7497^b^
Aurelia	2022	13.03^b^	9.78^a^	117^b^	0.13^a^	1040^ab^	3082^b^	298^b^	72.07^b^	2776^a^
Aurelia	2023	11.49^a^	9.25^a^	142^c^	0.26^b^	814^a^	1302^a^	216^a^	37.22^a^	3459^ab^
Cydora R.	2021	14.03^ab^	10.42^b^	59.30^a^	0.52^b^	1556^ab^	3183^b^	402^ab^	108.47^c^	4232^b^
Cydora R.	2022	15.95^b^	10.78^b^	191^b^	0.14^a^	1391^a^	2745^ab^	156^a^	79.12^b^	4234^b^
Cydora R.	2023	6.32^a^	9.37^a^	93.65^ab^	0.27^ab^	689^b^	705^a^	420^b^	45.53^a^	2147^a^
Hruškovitá	2021	9.73^a^	9.26	52.96^a^	0.27^b^	1305^b^	3433^ab^	226^a^	49.11^c^	4311^b^
Hruškovitá	2022	16.06^ab^	11.81	235^a^	0.15^a^	2222^c^	3814^b^	554^b^	44.14^b^	3568^a^
Hruškovitá	2023	16.84^b^	9.59	222^a^	0.26^b^	797^a^	1836^a^	1059^c^	31.97^a^	4067^b^
Izobilnaja	2021	15.05^b^	10.77^a^	69.48^a^	0.55^c^	817^a^	3374^b^	211^c^	26.54^ab^	6139^c^
Izobilnaja	2022	7.58^a^	11.12^a^	124^ab^	0.14^a^	1119^c^	2225^ab^	120^b^	61.89^b^	2727^a^
Izobilnaja	2023	9.15^ab^	11.08^a^	145^b^	0.30^b^	982^b^	871^a^	59^a^	18.15^a^	4033^b^
Kocúrova	2021	7.63^a^	10.04^a^	38.94^a^	0.52^b^	858	1572^b^	181^ab^	63.09^ab^	2305^a^
Kocúrova	2022	12.08^b^	11.12^b^	113^b^	0.14^a^	1141	2256^c^	99^a^	70.77^b^	3793^c^
Kocúrova	2023	11.25^ab^	9.44^a^	104^ab^	0.26	916	1154^a^	801^b^	37.70^a^	3356^b^
Konstantinopolska	2021	6.31^a^	9.08^a^	39.41^a^	0.49^b^	927^a^	1532^ab^	112^b^	51.60^a^	3745^c^
Konstantinopolska	2022	10.43^c^	11.44^c^	160^c^	0.15^a^	1564^c^	1951^b^	193^c^	52.34^a^	2535^b^
Konstantinopolska	2023	8.93^b^	9.86^b^	106^b^	0.26^ab^	1148^b^	697^a^	66^a^	30.75^a^	1808^a^
Morava	2021	16.86^a^	9.87^b^	89.31^a^	0.29^c^	2022^c^	5567^b^	445^b^	54.60^b^	4915^b^
Morava	2022	21.73^a^	10.54^c^	214^ab^	0.16^a^	1688^b^	5835^b^	531^c^	37.95^a^	4706^ab^
Morava	2023	22.20^a^	9.19^a^	253^b^	0.24^b^	974^a^	2556^a^	363^a^	45.07^ab^	3795^a^
Otličnica	2021	14.81^a^	10.08^b^	80.53^a^	0.26^b^	2141^c^	4556^b^	312^b^	39.62^ab^	4781^a^
Otličnica	2022	18.89^ab^	10.82^c^	249^b^	0.15^a^	0.00^a^	5049^c^	106^a^	32.08^a^	6285^b^
Otličnica	2023	32.28^b^	8.49^a^	436^c^	0.23^ab^	1102^b^	3439^a^	479^c^	86.78^b^	4992^ab^
Plovdivskaja	2021	15.34^c^	9.51^b^	86.42^a^	0.49^b^	2117^c^	5546^c^	507^c^	43.86^a^	4830^b^
Plovdivskaja	2022	13.00^b^	10.84^c^	169^c^	0.14^a^	1326^b^	3118^b^	380^b^	65.88^c^	3086^ab^
Plovdivskaja	2023	11.40^a^	7.54^a^	119^b^	0.21^ab^	818^a^	1706^a^	331^a^	51.05^b^	2270^a^

*Different letters indicate statistically significant differences (*p* < 0.05) among years within each cultivar, as determined by appropriate statistical

**Table 7:** Two-way ANOVA and effect size estimates for the influence of cultivar, year, and their interaction on phenolic compounds and antioxidant activity in quince peel

Variable	Factor	F	*p*-value	η²	ω²
TPC	Cultivar	1368.71	< 0.0001	0.583	0.583
	Year	161.83	< 0.0001	0.017	0.017
	C × Y	465.87	< 0.0001	0.397	0.396
DPPH	Cultivar	35.26	< 0.0001	0.230	0.223
	Year	287.52	< 0.0001	0.469	0.467
	C × Y	19.69	< 0.0001	0.257	0.244
FRAP	Cultivar	1399.58	< 0.0001	0.259	0.259
	Year	12858.51	< 0.0001	0.594	0.594
	C × Y	394.05	< 0.0001	0.146	0.145
ABTS	Cultivar	270.31	< 0.0001	0.078	0.078
	Year	10677.76	< 0.0001	0.775	0.775
	C × Y	249.09	< 0.0001	0.145	0.144
NCA	Cultivar	8243.73	< 0.0001	0.256	0.256
	Year	7536.59	< 0.0001	0.059	0.059
	C × Y	11025.41	< 0.0001	0.685	0.685
CA	Cultivar	2688.65	< 0.0001	0.451	0.451
	Year	10642.17	< 0.0001	0.446	0.446
	C × Y	302.25	< 0.0001	0.101	0.101
CCA	Cultivar	4176.37	< 0.0001	0.497	0.497
	Year	1028.06	< 0.0001	0.031	0.031
	C × Y	1982.65	< 0.0001	0.472	0.472
DCA	Cultivar	899.05	< 0.0001	0.346	0.346
	Year	1532.99	< 0.0001	0.148	0.147
	C × Y	654.35	< 0.0001	0.504	0.503
Rutin	Cultivar	686.81	< 0.0001	0.366	0.366
	Year	1451.26	< 0.0001	0.194	0.193
	C × Y	409.23	< 0.0001	0.437	0.435

*Degrees of freedom were identical for all variables (cultivar: 8; year: 2; interaction: 16). η² and ω² represent effect size estimates

Two-way ANOVA revealed that both cultivar and year significantly influenced total polyphenol content (TPC), antioxidant activity, and individual phenolic compounds (*p* < 0.0001 for all factors), with effect sizes indicating distinct patterns of variability among parameters (Table 7). Total polyphenol content (TPC) was predominantly driven by cultivar (η² = 0.583), with a substantial contribution of interaction effects (η² = 0.397), while the influence of year was minimal (η² = 0.017), indicating strong genetic control modulated by environmental conditions. In contrast, antioxidant activity assays showed method-dependent variability. DPPH activity was mainly influenced by year (η² = 0.469), while FRAP and ABTS were strongly dominated by year (η² = 0.594 and 0.775, respectively), indicating that antioxidant capacity is highly sensitive to environmental conditions, particularly annual variability. For individual phenolic compounds, distinct patterns were observed. Neochlorogenic acid exhibited a dominant interaction effect (η² = 0.685), indicating a strong dependence on the combined influence of cultivar and year. Chlorogenic acid showed a balanced contribution of cultivar (η² = 0.451) and year (η² = 0.446), suggesting comparable genetic and environmental control. Cryptochlorogenic acid was primarily influenced by cultivar (η² = 0.497) and interaction effects (η² = 0.472), while the effect of year was limited. Similarly, 3,5-dicaffeoylquinic acid was mainly governed by interaction effects (η² = 0.504), followed by cultivar (η² = 0.346), indicating a complex genotype–environment interplay. Rutin showed a more balanced contribution of cultivar (η² = 0.366), interaction (η² = 0.437), and year (η² = 0.194). It should be noted that, despite data transformation, ABTS values as well as neochlorogenic and chlorogenic acid concentrations did not fully meet the assumption of normality. Therefore, the ANOVA results for these variables should be interpreted with caution and primarily used to describe general variability patterns rather than precise statistical inference.

The variability observed in total polyphenol content, antioxidant activity, and individual phenolic compounds indicates a dynamic interaction between genetic background and environmental conditions. The strong cultivar effect observed for total polyphenol content suggests substantial genetic regulation of phenolic biosynthesis. Phenolic compounds are preferentially accumulated in peel tissues due to their protective role against oxidative stress, UV radiation, and pathogen attack. In contrast, antioxidant activity was more strongly influenced by year, indicating that environmental stress factors such as temperature fluctuations, drought, and solar radiation substantially affect antioxidant metabolism [35]. Significant cultivar × year interactions observed for several phenolic compounds further suggest that individual cultivars differed in their metabolic response and adaptability to environmental conditions during specific growing seasons (Table 8).

**Table 8:** Correlation matrix of phenolics and antioxidant activity (Spearman)

Variables	TPC	DPPH	FRAP	ABTS	NCA	CA	CCA	DCA	Rutin
TPC	**1**								
DPPH	0.01	**1**							
FRAP	**0.54**	0.20	**1**						
ABTS	-0.18	-0.36	**-0.69**	**1**					
NCA	0.25	0.33	-0.08	-0.08	**1**				
CA	**0.73**	0.16	0.08	-0.05	**0.58**	**1**			
CCA	**0.48**	-0.27	0.16	0.08	0.22	0.37	**1**		
DCA	0.00	-0.04	-0.14	-0.20	0.33	0.20	0.05	**1**	
Rutin	**0.69**	-0.03	0.01	0.27	0.26	**0.76**	0.19	-0.01	**1**

Values in bold are different from 0 with a significance level alpha=0.05

Spearman correlation analysis revealed distinct relationships among phenolic compounds and antioxidant activity parameters, reflecting differences in their chemical behavior and assay-specific responses. Total polyphenol content (showed strong positive correlations with chlorogenic acid and rutin, as well as a moderate correlation with cryptochlorogenic acid, indicating that these compounds are major contributors to the overall phenolic pool. Among individual phenolics, a particularly strong association was observed between chlorogenic acid and rutin. Neochlorogenic acid was moderately correlated with chlorogenic acid. In terms of antioxidant activity, FRAP exhibited a strong positive correlation with TPC, indicating that reducing power is closely linked to total phenolic content. In contrast, ABTS showed a strong negative correlation with FRAP and a moderate negative correlation with DPPH, highlighting substantial differences among antioxidant assays and their underlying mechanisms. DPPH displayed generally weak correlations with phenolic compounds, suggesting that radical scavenging activity measured by this assay is influenced by additional factors beyond total phenolic content. Similarly, dicaffeoylquinic acid showed only weak or negligible correlations with both phenolics and antioxidant parameters, indicating a more independent behavior within the phenolic profile. These results demonstrate that while total phenolic content is associated with key phenolic constituents such as chlorogenic acid and rutin, antioxidant activity varies depending on the assay used, reflecting differences in reaction mechanisms and sensitivity to specific compounds.

### Multivariate Analysis of Mineral Composition, Phenolic Profile, and Antioxidant Activity

To better understand the relationships between mineral composition, phenolic profile, and antioxidant activity in quince peel samples, a Spearman correlation analysis was performed (Table 9). This non-parametric approach was selected to evaluate potential associations between elements and bioactive compounds. Such analysis could provide insight into the underlying mechanisms linking elemental composition with phenolic metabolism and functional properties.

**Table 9:** Spearman correlation coefficients between mineral elements and antioxidant activity parameters and individual phenolic compounds in quince peel samples

Variables	TPC	DPPH	FRAP	ABTS	NCA	CA	CCA	DCA	Rutin
K	**0.44**	-0.05	**0.70**	**-0.63**	-0.02	0.21	0.06	0.15	0.02
Na	-0.21	0.33	-0.23	-0.01	0.22	0.09	-0.29	0.32	-0.03
Ca	-0.34	0.38	-0.34	0.14	0.29	-0.11	-0.33	0.25	-0.13
Mg	**0.41**	**0.46**	0.17	**-0.45**	**0.58**	**0.74**	0.11	**0.42**	0.30
P	**0.49**	**-0.46**	0.18	0.11	-0.08	0.34	0.19	0.01	**0.40**
Cu	0.26	**-0.39**	0.22	-0.02	0.08	0.11	0.28	-0.06	-0.08
Zn	0.30	-0.27	**0.62**	-0.28	-0.17	-0.08	0.23	-0.25	-0.10
Mn	-0.22	**0.56**	-0.22	0.09	0.32	-0.01	-0.27	0.28	-0.06
Fe	0.25	-0.26	0.30	-0.01	-0.04	0.11	0.23	-0.04	0.08
Cr	0.16	**0.67**	0.32	**-0.59**	0.27	0.24	-0.15	0.23	-0.17
Ni	-0.02	**0.46**	**0.64**	**-0.80**	-0.03	-0.22	-0.14	0.08	**-0.46**
Co	0.25	**0.47**	0.19	**-0.56**	0.31	**0.55**	-0.16	0.33	0.17
Pb	-0.27	**0.47**	0.16	-0.28	0.04	-0.26	-0.20	0.05	-0.32
Cd	-0.16	0.36	-0.16	-0.13	0.38	0.05	-0.24	0.31	-0.12

*Values in bold are different from 0 with a significance level alpha = 0.05

**Fig. 1:**
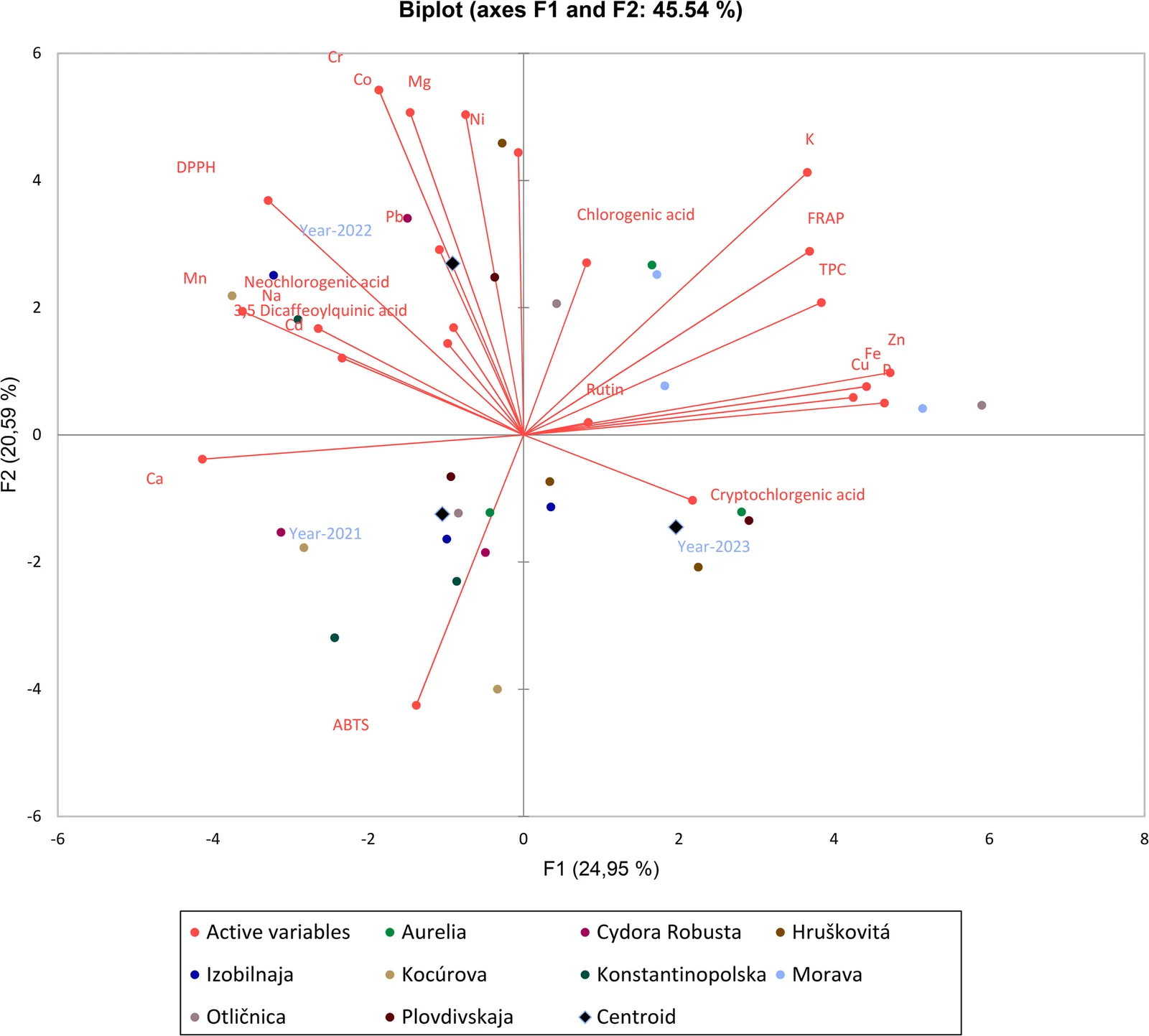
Principal component analysis (PCA) biplot showing the relationships among mineral elements, total polyphenol content, antioxidant activity, and individual phenolic compounds in quince peel samples

Spearman correlation analysis revealed significant relationships between mineral elements and both phenolic composition and antioxidant activity, indicating a potential link between mineral nutrition and secondary metabolism in quince peel. Among macronutrients, potassium showed a strong positive correlation with FRAP and a moderate positive correlation with TPC, while exhibiting a strong negative correlation with ABTS. Similarly, phosphorus was positively associated with TPC and rutin but negatively correlated with DPPH. Magnesium demonstrated a particularly strong relationship with phenolic compounds, showing high positive correlations with chlorogenic acid and neochlorogenic acid, as well as moderate associations with TPC and dicaffeoylquinic acid. In addition, cobalt was positively correlated with chlorogenic acid. Among microelements, zinc and nickel exhibited strong positive correlations with FRAP. In contrast, ABTS showed strong negative correlations with several elements, particularly nickel, chromium, cobalt, and potassium. DPPH was positively correlated with several elements, including manganese, chromium, nickel, cobalt, and lead. To explore the multivariate structure of the dataset and identify underlying patterns among mineral elements, phenolic compounds, and antioxidant activity parameters, principal component analysis (PCA) was performed (Fig. 1). This approach was applied to reduce data dimensionality and to reveal relationships, clustering trends, and the main sources of variability among samples. PCA enables the identification of key variables contributing to sample differentiation and provides a comprehensive overview of the interactions between elemental composition and bioactive compounds. The multivariate analysis confirmed a strong interplay between mineral composition, phenolic profile, and antioxidant activity in quince peel samples. Principal component analysis (PCA) revealed that the first two components explained 45.54% of the total variance, with PC1 representing a nutrient-driven bioactive axis and PC2 reflecting differences in antioxidant mechanisms and trace element influence. PC1 was positively associated with essential elements (Zn, Fe, Cu, P, K) together with total polyphenol content and FRAP activity, indicating that mineral-rich samples exhibited enhanced reducing antioxidant capacity. In contrast, Ca and Mn were negatively associated with this axis. PC2 was dominated by transition elements (Cr, Co, Ni, Mg) and DPPH activity, while ABTS showed an opposite trend, confirming that different antioxidant assays capture distinct reaction mechanisms. The third principal component further highlighted variability in phenolic composition, with strong contributions from chlorogenic acid, rutin, and other caffeoylquinic acid derivatives, indicating that differences among samples are driven not only by total phenolic content but also by qualitative composition. These findings were consistent with the correlation analysis, which demonstrated strong positive relationships between TPC, FRAP, and key phenolic compounds, as well as significant associations between mineral elements and antioxidant activity. Notably, clear cultivar-dependent patterns were observed, with Morava and Otličnica characterized by higher bioactive potential, while Konstantinopolska and Cydora Robusta exhibited lower values.

## Conclusion

Quince peel, representing a major by-product of technologically processed quince fruits, demonstrated a significant content of essential mineral elements, phenolic compounds, and antioxidant activity, indicating its potential as a valuable plant-based raw material for food applications. The investigated quince cultivars exhibited considerable variability in mineral composition, antioxidant activity, and phenolic profile depending on cultivar, year of cultivation, and their interaction, highlighting the combined influence of genetic and environmental factors. The obtained results indicate that quince peel may represent a promising source of minerals and phenolic compounds with potential applicability in the development of enriched or innovative food products. Nevertheless, further studies focused on bioaccessibility, technological functionality, sensory properties, and stability during food processing are necessary to fully evaluate its practical utilization in food systems.

It is estimated that quince, as a fruit, produces up to 50% of by-products during processing, of which peels and pomace constitute the largest proportion. These by-products have been used usually in agriculture as animal feed, fertilizer or in methane fermentation. In general, these by-products often contain biologically valuable compound for food industry. The results of this study indicated the possibility of using the quince peel as a source of important minerals in the production of functional foods. At the same time, the peels can be considered as a valuable raw material with promising potential for its use in the development of new value-added foods due to its remarkable content of phenolic compounds such as chlorogenic acid, or rutin, that have several positive effects on human health. Multivariate analysis further confirmed that the chemical composition of quince peel is strongly shaped by both genetic and environmental factors. This highlights the importance of considering genotype–environment interactions when evaluating the nutritional and functional potential of plant-derived materials. Moreover, the observed correlations between mineral elements and bioactive compounds suggest that mineral composition may play a role in modulating phenolic biosynthesis and antioxidant capacity.

By utilizing fruit processing waste, nutritional and health benefits can be achieved in the form of innovative food products and enriched functional foods at minimal cost, while minimizing the generation of biowaste. Utilization of fruit and vegetable processing waste is an effective tool for supporting sustainable development. By-products of fruit and vegetable processing will find their application in various new food recipes, in the design of novel foods with enhanced mineral and antioxidant properties, especially in innovative bakery products (as fiber and antioxidant-rich flour substitutes), jams (as natural thickening and phenolic-enriching agents), and meat products (as natural antioxidants and functional extenders).

For practical application, it is recommended that the selection of quince peel raw material for food formulation should be based on a combination of key indicators, including selected macro- and microelement contents, major phenolic compounds, and antioxidant activity, rather than relying on individual parameters alone, as this approach provides a more robust basis for decision-making in the development of functional food products.

## Supplementary Information

Below is the link to the electronic supplementary material.

Supplementary Material 1

## Data Availability

No datasets were generated or analysed during the current study.
