# Supplementary material for: Mineral Content and Bioactive Potential of Quince (Cydonia oblonga) Peels for Value-Added Food Production
Source: Plant Foods Hum Nutr. 2026 Jun 18;81(3):78. doi: 10.1007/s11130-026-01528-7 (PMC13279378; doi:10.1007/s11130-026-01528-7)
Supplement: Supplementary file 1 — Supplementary Material 1 [file 11130_2026_1528_MOESM1_ESM.docx]

**SUPPLEMENTARY MATERIAL**

**Mineral content and bioactive potential of quince (*Cydonia oblonga*) peels for value-added food production**

VOLLMANNOVA Alena, TOTH Tomas, LIDIKOVA Judita, MUSILOVA Janette, CERYOVA Natalia, JAKUBCINOVA Jana*

Institute of Food Sciences, Faculty of Biotechnology and Food Sciences, Slovak University of Agriculture in Nitra, Tr. A. Hlinku 2, 94976 Nitra, Slovakia

*Corresponding author: jana.jakubcinova@uniag.sk

**Material and Methods**

**Plant material**

Nine selected quince varieties (Aurelia, Cydora Robusta, Hruškovitá, Izobilnaja, Kocúrova, Konstantinopolska, Morava, Otličnica, and Plovdivskaja) were obtained from the organic farm Blatnička (Czech Republic) and were analyzed over a three-year period.

Aurelia – hard, aromatic fruits with a smooth yellow-green skin.

Cydora Robusta – large, fragrant and bright yellow fruits with a medium-thick yellow skin.

Hruškovitá – deep yellow, pear-shaped, plump fruits.

Izobilnaja – fruits of a typical wide, pear-shaped shape, lemon-yellow colour, hard, very aromatic, skin.

Kocúrova – pear-shaped fruits with deep yellow skin, with a distinctive aroma and taste.

Konstanopolska – apple-shaped fruits with a distinctive aroma, light yellow flesh, juicy, delicate, aromatic.

Morava – large fruits with a velvety surface and an intense yellow colour when ripe.

Otličnica – large fruits with a firm bright yellow skin.

Plovdivskaja – large fruits with yellow skin and pale-yellow flesh.

Quince fruits were harvested during the 2021–2023 growing seasons at technological maturity characterized by fully developed yellow skin coloration and typical varietal aroma. For each cultivar, fruits were collected from 3 independent trees, and each biological replicate consisted of 3 fruits randomly collected from different parts of the canopy.

Organic Farm Blatnička is a small organic farm in the foothills of the White Carpathian Mountanis close to the Czech-Slovak border. The Blatnička farm focuses primarily on the production of old forgotten varieties of fruit or other traditional crops, which are now considered to be forgotten plant species. Many of them are being rediscovered today due to their unique composition and content of biologically valuable components, and there is currently a growing interest in their use in innovative food products. For example, minority traditional fruit species such as chokeberry, dogwood, hawthorn, medlar, rosehip, rowan, mulberry or quince are grown in Blatnička. In its eco-orchards in South Moravia, the Blatnička farm currently grows 39 varieties of quince.

**Sample preparation**

After sampling the quince fruits, the fruits were cleaned in distilled water and then dried on filter paper at room temperature (22°C). The quince fruits were peeled, and the peels were used to prepare the extract. Fresh quince peel material was used for extraction.

**Extract preparation**

All quince peel samples were homogenized (Grindomix GM 200, Retsch GmbH, Germany) and 25 g of the homogenized material was extracted with 50 mL of 80% methanol for 12 h on a horizontal shaker (Heidolph Promax 1020, Heidolph Instruments GmbH, Germany) at 115 oscillations per minute. After shaking, the extracts were filtered through Munktel No. 392 filter paper (Munktell & Filtrac GmbH, Germany). The extracts prepared in this way were stored in sealable centrifuge tubes at 4°C in a refrigerator until analysis.

**Chemical analysis**

*Content of macroelements*

Dried samples (1 g) were digested with 10 mL concentrated HNO_3_ and 5 mL concentrated HClO_4_. The mixtures were left to stand overnight and subsequently mineralized in a sand bath until the evolution of white fumes indicated complete digestion. After cooling, the digests were filtered into 100 mL volumetric flasks, and the filter paper was rinsed with hot redistilled water. The solutions were then brought to volume with redistilled water. Reagent blanks were prepared following the same procedure. Prior to analysis, an aliquot (2 mL) of each digest was diluted to 50 mL with redistilled water. Elemental concentrations were determined by flame atomic absorption spectrometry (AAS) using a Varian AA 240FS. The following wavelengths were used: K (766.5 nm), Ca (422.7 nm), Na (589.0 nm), and Mg (285.2 nm) **[1,2]**.

Phosphorus was determined using the molybdenum blue method **[3]**. An aliquot of the digest (1 mL) was transferred to a 50 mL volumetric flask, mixed with 8 mL of colour reagent (Solution A), and diluted to volume with redistilled water. The mixture was allowed to stand for 2 h at room temperature to ensure full colour development. Absorbance was then measured at 666 nm using a Shimadzu UV-1800. Solution A (colour reagent) was prepared by dissolving 2.64 g of ascorbic acid in a mixture containing 500 mL of Solution B and diluting to 2 L with redistilled water. Solution B consisted of 148 mL concentrated H_2_SO_4_, 12 g ammonium molybdate ((NH_4_)_2_MoO_4_), and 0.2908 g potassium antimonyl tartrate, diluted to volume with redistilled water.

*Content of microelements*

Dried samples were homogenized, and 1 g of each sample was subjected to microwave-assisted digestion using a mixture of 5 mL redistilled water and 5 mL extra-pure concentrated HNO_3_ (1:1, v/v). Digestion was performed in sealed vessels using a CEM MARS Xpress. The digestion program (total time 55 min) consisted of three steps: ramp to 160 °C over 15 min at 800 W (90% power), hold at 160 °C for 20 min, and cooling.

After digestion, the solutions were filtered through quantitative filter paper (Munktell grade 390) into 50 mL volumetric flasks and diluted to volume with redistilled water.

Elemental analysis was carried out by atomic absorption spectrometry using a Varian AA 240FS/240Z. Copper (324.8 nm), zinc (213.9 nm), manganese (279.5 nm), iron (248.3 nm), chromium (357.9 nm), nickel (232.0 nm), and cobalt (240.7 nm) were determined by flame AAS, while lead (217.0 nm) and cadmium (228.8 nm) were quantified using graphite furnace AAS with Zeeman background correction **[1,4]**.

Quality assurance and quality control procedures were applied to ensure the accuracy and reliability of the analytical results. Calibration curves were prepared using standard solutions (Merck, Germany) over appropriate concentration ranges for each element. The linearity of calibration was confirmed with correlation coefficients (R^2^) higher than 0.999 for all analytes. Analytical accuracy was verified using a certified reference material (CRM; ERM-CD281 Rye Grass, Institute for Reference Materials and Measurements, Geel, Belgium), which was processed and analyzed under the same conditions as the samples in six replicates. Recovery values for the analyzed elements ranged from 91.7% to 106.0% indicating good agreement with certified values and confirming method accuracy. Repeatability of the analytical method was evaluated based on replicate measurements and expressed as relative standard deviation (RSD), which did not exceed 3% for any element. For elements not included in the CRM, accuracy was ensured through calibration, repeatability assessment, and consistency of analytical performance. Procedural blanks were included in each batch of analyses and measured n triplicate to monitor potential contamination; blank values were negligible and subtracted where necessary. Limits of detection (LOD) and limits of quantification (LOQ) were determined as threefold and ninefold the standard deviation of the blank signal, respectively, and are reported in the corresponding sections. LOD for Pb and Cd was 0.1 and 0.06 µg/L, LOQ for Pb and Cd was 0.3 and 0.18 µg/L respectively

*Total polyphenol content*

Total polyphenol content (TPC) was determined using the Folin–Ciocalteu method **[5]**. An aliquot of the methanolic extract (0.1 mL) was mixed with 2 mL of distilled water in a 50 mL volumetric flask, followed by the addition of Folin–Ciocalteu reagent. After 3 min, 5 mL of 20% (w/v) sodium carbonate solution was added, and the mixture was diluted to volume with distilled water. The reaction mixture was incubated for 2 h at room temperature to allow the formation of a blue-coloured complex.

Absorbance was measured at 765 nm using a PG Instruments T92+ against a reagent blank. A calibration curve was constructed using gallic acid as the standard, and results were expressed as mg gallic acid equivalents per g of dry weight (mg GAE/g DW). All measurements were performed in quadruplicate.

*Antioxidant activity*

***DPPH radical scavenging assay***

Antioxidant activity was evaluated using the DPPH radical scavenging method **[6]**. A solution of 0.06 mM DPPH• (2,366 mg in 100 mL methanol, 99.8%) was prepared and stored at 4 °C in the dark. The initial absorbance (A₀) of the DPPH solution was measured at 515.6 nm using a PG Instruments T92+.

An aliquot of the extract (0.1 mL) was added to the DPPH• solution, and the absorbance (A₁₀) was recorded after 10 min of incubation in the dark. Results were quantified using a Trolox calibration curve and expressed as mmol Trolox equivalents per kg dry weight (mmol TE/kg DW). All measurements were performed in quadruplicate.

***ABTS radical scavenging assay***

The ABTS assay was performed according to Re et al. **[7]**. The ABTS•⁺ radical cation was generated by reacting

7 mM ABTS (2,2′-azinobis-(3-ethylbenzothiazoline-6-sulfonic acid)) with 2,45 mM potassium persulfate and

diluted in acetate buffer (pH 4.5) to obtain the ABTS•⁺ solution. A volume of 3 mL of ABTS•⁺ solution was mixed

with 0.05 mL of extract, incubated in the dark for 20 min, and absorbance was measured at 734 nm using a Shimadzu UV-1800.

Antioxidant activity was calculated from a Trolox calibration curve and expressed as mmol TE/kg. DW. All

analyses were performed in quadruplicate.

***FRAP assay***

Ferric reducing antioxidant power (FRAP) was determined according to Benzie and Strain **[8]**. The FRAP reagent was prepared from 10 mM TPTZ (2,4,6-tris(2-pyridyl)-s-triazine), 20 mM FeCl_3_, and acetate buffer (pH 3.6) and incubated in 37°C for 10 minutes.

A mixture of 3 mL FRAP reagent and 0.05 mL extract was incubated in in 37°C for 4 minutes, and absorbance was measured at 593 nm using a Shimadzu UV-1800. Antioxidant activity was calculated using a Trolox calibration curve and expressed as mmol TE/kg DW. All measurements were conducted in quadruplicate.

*Content of selected phenolics*

Before HPLC analysis, the prepared methanol extracts were filtered through a Q-Max syringe filter (0.22 μm, 25 mm, PVDF) (Frisenette ApS, Knebel, Denmark) into HPLC vials.

The content of individual selected compounds was determined using an Agilent 1260 Infinity II HPLC high-performance liquid chromatograph (Agilent Technologies, Waldbronn, Germany) with a diode array detector. The quantification wavelength was set to 320 nm for chlorogenic, cryptochlorogenic, neochlorogenic and 3,5- dicaffeoylquinic acid and to 372 nm for rutin. The content of the determined compounds was expressed in mg/kg DW.

Chromatographic separation was performed using a CORTECS C18 column (150 × 4.6 mm, 2.7 μm). The mobile phase consisted of (A) 0.1% phosphoric acid in water, (B) acetonitrile, and (C) methanol under gradient elution conditions. The flow rate was 1.0 mL/min and the injection volume was 8 μL. The quantification wavelength was set to 320 nm for chlorogenic, cryptochlorogenic, neochlorogenic and 3,5- dicaffeoylquinic acid and to 372 nm for rutin. The content of the determined compounds was expressed in mg/kg DW. Identification of compounds was based on retention times and UV spectra of analytical standards (Sigma Aldrich, Saint-Louis, Missouri, USA). Calibration curves showed excellent linearity (R² > 0.999). LOD values for chlorogenic, cryptochlorogenic, neochlorogenic, 3,5-dicaffeyolqunic acid, and rutin were 0.22, 0.81, 1.08, 0.81, 1.09 µg/mL respectively, and LOQ values were 0.67, 2.54, 3.36, 2.44, and 3.95 µg/mL respectively.

The HPLC-DAD method was verified in terms of linearity, limits of detection and quantification, precision, repeatability, and recovery. Calibration curves were prepared using authentic standards of neochlorogenic acid, chlorogenic acid, cryptochlorogenic acid, 3,5-dicaffeoylquinic acid, and rutin at six concentration levels. Linearity was evaluated by plotting peak area against standard concentration. LOD and LOQ were calculated as 3.3σ/S and 10σ/S, respectively, where σ is the standard deviation of the response and S is the slope of the calibration curve. Precision was assessed by repeated injection of the same standard solution, while repeatability was evaluated by repeated preparation and analysis of the same quince peel extract. Precision expressed as RSD was below 3%. Accuracy was assessed by recovery experiments using quince peel extracts spiked with known concentrations of analytical standards.

**Statistical analysis**

Data are presented as mean ± standard deviation of three independent replicates. Statistical analyses were performed using XLSTAT **[9].** All statistical tests were evaluated at a significance level of α = 0.05. Prior to analysis, the normality of data distribution was evaluated using the Shapiro–Wilk test, and homogeneity of variances was assessed using Levene’s test. For parameters that met the criteria of normal distribution, differences in individual cultivars among years were evaluated using one-way analysis of variance (ANOVA). When significant effects were detected, Tukey’s honestly significant difference (HSD) test was applied for post hoc multiple comparisons. For datasets that did not meet the assumption of normality, a non-parametric Kruskal Wallis test was employed. In cases where significant differences were observed, pairwise comparisons were conducted using Dunn’s test with Holm–Bonferroni correction to control for type I error. As several variables exhibited non-normal distribution, data were log-transformed [log_10_(x + 1)] to improve normality and homoscedasticity. Despite transformation, selected variables (Pb, Cd, Cr, Ni, Co, ABTS, neochlorogenic acid, and chlorogenic acid) did not fully meet the assumption of normality; therefore, the results for these variables were interpreted with caution. Nevertheless, given the balanced experimental design, ANOVA was considered robust to moderate deviations from normality and was used primarily to describe overall variability patterns. The effects of cultivar, year, and their interaction were evaluated using two-way analysis of variance (ANOVA). Effect sizes were expressed as eta squared (η²) and omega squared (ω²), enabling quantification of the proportion of explained variance. Correlation analysis was performed using Spearman’s rank correlation coefficient to evaluate relationships between mineral elements, phenolic compounds, and antioxidant activity parameters, due to the non-normal distribution of several variables. Principal component analysis (PCA) was conducted to explore multivariate relationships and identify patterns in the dataset. Variables were standardized prior to analysis, and principal components with eigenvalues greater than 1 were retained for interpretation. PCA results were visualized using biplots to assess the relationships between samples and variables.

1. Welz B, Sperling M (1999) *Atomic absorption spectrometry*. Wiley-VCH, Weinheim. <https://doi.org/10.1002/9783527611690>
2. AOAC International (2019) *Official methods of analysis of AOAC International*, 21st edn. AOAC International, Rockville, MD.
3. Murphy J, Riley JP (1962) A modified single solution method for the determination of phosphate in natural waters. *Anal Chim Acta* 27:31–36. <https://doi.org/10.1016/S0003-2670(00)88444-5>
4. United States Environmental Protection Agency (USEPA) (1996) *Test methods for evaluating solid waste, SW-846. Method 3052: Microwave-assisted acid digestion of siliceous and organically based matrices*. U.S. EPA, Washington, DC.
5. Singleton VL, Orthofer R, Lamuela-Raventós RM (1999) Analysis of total phenols and other oxidation substrates and antioxidants by means of Folin-Ciocalteu reagent. In: Packer L (ed) Methods in enzymology, vol 299. Academic Press, San Diego, pp 152–178. <https://doi.org/10.1016/S0076-6879(99)99017-1>
6. Brand-Williams W, Cuvelier ME, Berset C (1995) Use of a free radical method to evaluate antioxidant activity. *LWT Food Sci Technol* 28:25–30. <https://doi.org/10.1016/S0023-6438(95)80008-5>
7. Re R., Pelegrini, N, Proteggente, A, Pannala, A, Yang, M, Rice-Evans, C (1999). Antioxidant activity

applying an improved abts radical cation decolorization assay. *Free Radical Biology & Medicine,*vol. 26, nos. 9/10, pp. 1231–1237. [https://doi.org/ 10.1016/s0891-5849(98)00315-3](https://doi.org/10.1016/S0023-6438(95)80008-5).

1. Benzie IFF, Strain JJ (1996) The ferric reducing ability of plasma (FRAP) as a measure of “antioxidant power”: The FRAP assay. *Anal Biochem* 239:70–76. <https://doi.org/10.1006/abio.1996.0292>
2. Lumivero (2026) XLSTAT statistical and data analysis solution (Version 2026.1) [Computer software]. <https://www.xlstat.com>
